# Arrhythmogenicity of anti-tachycardia pacing in patients with implantable cardioverter defibrillator

**DOI:** 10.1186/s43044-023-00369-y

**Published:** 2023-06-02

**Authors:** Sherien Samy Awad, Emmanuel Fares Azeez, Mohamed Ossama Taha, Wael Mohamed El-Naggar, Ahmed El-Damaty

**Affiliations:** 1 Egyptian Ministry of Health, Al Kasr Al Aini Street, Old Cairo, 11562 Cairo Governorate Egypt; 2 Cairo University, Cardiovascular Medicine, Cairo, Egypt; 3 National Heart Institute, Cardiovascular Medicine, Cairo, Egypt

**Keywords:** ATP therapy, VT acceleration, Ramp, Scanning, Ventricular tachycardia

## Abstract

**Background:**

Anti-tachycardia pacing therapy (ATP) has shown comparable efficacy to shock therapy in ventricular tachycardia (VT) termination with better quality of life. However, some ATPs may lead to VT acceleration or degeneration to ventricular fibrillation (VF), which will result in more ICD shocks. The aim of this study was to investigate the predictors of VT acceleration by ATP therapy in a real-life patient cohort.

**Results:**

We retrospectively reviewed 448 monomorphic VT episodes that required ATP therapy in 60 patients with structural heart diseases implanted with ICD or CRTD. The clinical data of the patients and the episodes’ details were evaluated. We found that patients with a higher ejection fraction (EF) were more likely to be cardioverted by ATP therapy (*P*: 0.024). VT acceleration was more frequent in patients with lower EF (mean 31.24 ± 4.08) compared with the non-accelerated patients with higher EF (mean 37.00 ± 9.4, *P*: 0.016). The percentage of accelerated episodes was 8.5%. VT episodes with a mean cycle length (CL) < 310 ms are more likely to accelerate (sensitivity 76.3%, specificity 67.7%, PPV value 45%, NPV 86%, and AUC 0.790). There was a statistically significant difference in the accelerated VT episodes as compared to non-accelerated episodes regarding the number of ATP bursts (mean 3.66 ± 2.22 vs. 1.76 ± 1.35, *P*: < 0.001), ramp (23.7% vs. 4.2%, *P*: < 0.001), scanning (55.3% vs. 31.3%, *P*: 0.003) and burst adaptive cycle length (mean 83.55 ± 2.92 vs. 84.64 ± 2.61, *P*: 0.016). In a multivariate analysis, the VT CL, number of ATP bursts and ramp pacing predicted VT acceleration by ATP therapy.

**Conclusions:**

Ventricular tachycardia in patients with low LV EF and fast VTs with a CL less than 310 ms were more likely to accelerate with ATP therapy. The number of ATP bursts and the use of ramp had a significant effect on VT acceleration. To avoid VT acceleration by ATP therapy, ramp pacing better be avoided, especially in fast VTs, and lesser number of bursts should be delivered.

## Background

Implantable cardioverter defibrillators (ICDs) offer a well-proven mortality benefit among patients at high risk of sudden arrhythmic death [1]. ICDs provide two types of therapy: high-energy shocks and anti-tachycardia pacing (ATP). Shock-induced electroporation and incapacitation is the proposed anti-fibrillatory mechanism for tachycardia termination, which is efficient; however, it is associated with a pro-fibrillatory effect that includes transient ectopy, tachycardia, bradycardia, complete heart block, increased pacing threshold as well as atrial and ventricular mechanical dysfunction due to transient muscle damage [2]. Despite that survival benefit, fears from receiving ICD shocks have been identified as a major determinant of psychological stress, anxiety, depression in addition to a proven association with left ventricular dysfunction [3]. ATP therapy uses a low energy patterned stimulation to terminate ventricular tachycardia (VT), it has programmable options, it is painless and effective in monomorphic VT, especially the slow ones, but not effective in polymorphic VT or ventricular fibrillation (VF) termination. On the other hand, ATP also might accelerate the tachycardia or cause degeneration to VF [4]. The aim of the study is to evaluate the pro-arrhythmic effect of anti-tachycardia pacing (ATP) and its predictors in patients with implantable cardioverter defibrillators in a real-life patient’s cohort.

## Methods

We retrospectively reviewed patients data with ICDs presenting to the outpatient arrhythmia and pacemaker follow-up clinic at two tertiary care centers, over a period of 24 months, who received ATP therapy for terminating monomorphic VT episodes. The study complied with the Declaration of Helsinki, and the study protocol was approved by the local research committee. All patients signed an informed written consent. The following patients’ groups were excluded from the study: (i) patients without ATP therapies during the follow-up, (ii) patients with polymorphic VT/VF episodes as determined by the intracardiac far-field EGM, (iii) patients who received inappropriate ATP therapy for supraventricular tachycardia or lead problems, (iv) patients with episodes with no stored EGMs, for example, episodes exceeding the storage of device memory or non-sustained episodes.

The following data were collected from all eligible patients: demographic data including age and gender, clinical data including: preimplantation clinical diagnosis, e.g., (ischemic cardiomyopathy, dilated cardiomyopathy, arrhythmogenic right ventricular cardiomyopathy and hypertrophic cardiomyopathy), the indication of implantation (primary or secondary prevention of sudden cardiac death), previous transthoracic echocardiography for assessment of the ejection fraction by Simpson’s method and the patients’ medical treatment including antiarrhythmic medications received during the analyzed episodes period.

All available stored episodes were retrieved from the programmers during the active session and collected as PDF files on a USB flash drive that can be viewed on a computer for further analysis. These episodes were analyzed regarding the VT cycle length and morphology, ATP therapy programming parameters and response to ATP therapy. Episodes defined as VT by the device discriminators were revised for inappropriate detection; ventricular rates exceeding 220 Bpm were labeled as VF. The VT morphology (monomorphic vs. polymorphic) was determined based on the intracardiac far-field electrograms. The VT CL was the mode of the VT CL in the last 15 RR intervals before the first ATP delivery, and the mean VT CL was the mean of the VT CL in the last 15 RR intervals before the first ATP delivery. The response to ATP therapy was classified as (i) VT acceleration: defined as a decrease in the VT CL by > 10% or VT degeneration to ventricular flutter or VF after the ATP attempt, (ii) ATP therapy success: defined as VT termination and return to sinus rhythm after the ATP attempt, (iii) ATP therapy failure: defined as persistence of VT after ATP attempt that ended by HV defibrillation, spontaneous termination or VT deceleration below the detection rate. Patients were classified into two groups according to their response to the ATP therapy, the accelerated versus the non-accelerated group; at least one accelerated episode was required for a patient to be assigned to the accelerated group.

### Statistical analysis

The data were analyzed using the Statistical Package for Social Sciences, version 20.0 (SPSS Inc.). Qualitative data were expressed as percentage and frequency. Independent samples (t test) of significance was used for comparing between two means. Chi-square (χ^2^) test of significance was used in order to compare proportions between qualitative parameters. Quantitative data were expressed as mean ± standard deviation (SD). Binary logistic regression was used to predict the outcome of categorical variable based on multiple predictor variables. Receiver operating characteristic (ROC curve) analysis was used to define a cutoff value of VT cycle length that predicts VT acceleration. *P*-value < 0.05 was considered significant.

## Results

Among 315 patients with ICDs reviewed in the outpatient clinic, sixty patients received ATP therapy for sustained monomorphic VT. The demographic and clinical characteristics of the study population are shown in Table 1. The retrieved VT episodes in 60 patients were 980 episodes; 448 received ATP episodes with a mean VT CL 335.73 ± 51.22 ms.

**Table 1:** Baseline characteristics of the study population (*n* = 60)

Baseline characteristics	Number (%)
Sex	
Female	6 (10.0%)
Male	54 (90.0%)
Age (years)	
Range	38–80
Mean ± SD	56.60 ± 9.44
Diagnosis	
ARVD	2 (3.3%)
DCM	16 (26.7%)
HOCM	1 (1.7%)
ICM	41 (68.3%)
ICD indication	
Primary prevention	4 (6.7%)
Secondary prevention	56 (93.3%)
Device type	
CRTD	15 (25.0%)
Single-chamber ICD	22 (36.7%)
Dual-chamber ICD	23 (38.3%)
EF%	
Range	21–62
Mean ± SD	35.03 ± 7.72

*N* Number, *SD* standard deviation, *ARVD* arrhythmogenic right ventricular cardiomyopathy, *DCM* dilated cardiomyopathy, *HOCM* hypertrophic obstructive cardiomyopathy, *ICM* ischemic cardiomyopathy, *CRTD* cardiac resynchronization therapy with defibrillator, *ICD* implantable cardioverter defibrillator, *EF* ejection fraction

The number of bursts delivered in each ATP therapy ranged from 1 to 11 bursts (mean = 1.92 ± 1.54 SD); the number of stimuli in each delivered burst ranged from 5 to 13 stimuli (mean = 8.24 ± 1.05 SD) with an adaptive cycle length ranging from 75 to 88% (mean = 84.54 ± 2.65 SD). Scan was on, in 149 episodes (33.3%) with a scan step − 10 ms in all those episodes. Ramp was on, in a lesser percentage of episodes, 26 episodes (5.8%).

Off the 448 analyzed episodes, ATP therapy was successful in terminating the VT episodes in 313 episodes (69.86%); the remaining episodes either accelerated to a faster VT or degenerated to VF to be terminated by high-voltage (HV) therapy in 38 episodes (8.48%) or failed to be terminated by ATP therapy in 97 episodes (21.65%). Failure of ATP therapy led to VT termination by HV therapy in 88 episodes (19.64%) or deceleration of the VT below the detection rate in 6 episodes (1.33%) and spontaneous termination in 3 episodes (0.66%).

When comparing patients who had VT acceleration (17 patients) and patients who did not (43 patients) according to their demographic and clinical data, there was no statistical difference in the age and sex, the preimplantation diagnosis, indication for implantation or antiarrhythmic drug therapy. Among the patients’ clinical data, the left ventricle ejection fraction as measured by Simpson’s method showed a statistically significant difference, being lower in the accelerated group compared to the non-accelerated group, respectively [31.24 ± 7.08 vs. 37.00 ± 9.04, *P* value: 0.016] Table 2.

**Table 2:** Comparison between ATP-accelerated group and ATP non-accelerated group according to baseline characteristics

Baseline characteristics	ATP accelerated group (*n* = 17)	ATP non-accelerated group (*n* = 43)	Test	*P*-value
Sex				
Female	2 (11.8%)	4 (9.3%)	*χ*^2^ = 0.082	0.774
Male	15 (88.2%)	39 (90.7%)		
Age (years)				
Mean ± SD	56.59 ± 9.31	56.60 ± 9.60	0.126	0.895
Range	39–72	38–80		
Diagnosis				
ARVD	1 (5.9%)	1 (2.3%)	*χ*^2^ = 3.733	0.292
DCM	3 (17.6%)	13 (30.2%)		
HOCM	1 (5.9%)	0 (0.0%)		
ICM	12 (70.6%)	29 (67.4%)		
Indications				
Primary	1 (5.9%)	3 (7.0%)	*χ*^2^ = 0.023	0.878
Secondary	16 (94.1%)	40 (93.0%)		
AAD therapy				
BB	5 (29.4%)	17(39.5%)	*χ*^2^ = *8.266*	0.142
BB + Amiodarone	8 (41.2%)	13(16.3%)		
BB + Ivabradine	0 (0.0%)	3 (7.0%)		
Amiodarone + Ivabradine	0 (0.0%)	2 (2.3%)		
BB + Amiodarone + Ivabradine	1 (0.0%)	1 (2.3%)		
BB + Sotalol	2 (5.9%)	0 (0.0%)		
BB + Amiodarone + Sotalol	0 (0.0%)	3 (2.3%)		
Amiodarone	0 (0.0%)	1 (2.3%)		
Digoxin	0 (0.0%)	1 (2.3%)		
Ivabradine + Sotalol	1 (5.9%)	0 (0.0%)		
Ivabradine + Digoxin	0 (0.0%)	1 (2.3%)		
Sotalol	0 (0.0%)	1 (2.3%)		
EF%				
Mean ± SD	31.24 ± 7.08	37.00 ± 9.04	2.565	0.016*
Range	21–55	24–62		

*ATP* anti-tachycardia pacing, *N* number, *SD* standard deviation, *ARVD* arrhythmogenic right ventricular cardiomyopathy, *DCM* dilated cardiomyopathy, *HOCM* hypertrophic obstructive cardiomyopathy, *ICM* ischemic cardiomyopathy, *AAD* antiarrhythmic drug therapy, *BB* betablockers, *EF* ejection fraction

Using: t-independent sample t test; χ^2^: Chi-square test. *P*-value > 0.05 NS; *P-value < 0.05 S; **P-value < 0.001 HS

**Fig. 1:**
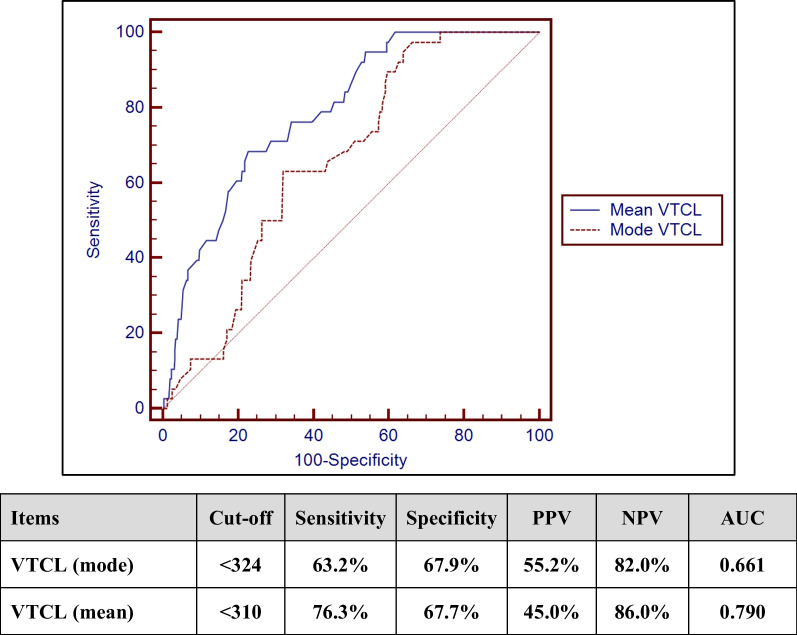
Diagnostic performance of VTCL (mode) and mean VTCL in discriminating VT acceleration by ATP therapy

When comparing the accelerated VT episodes (38 episodes) and the non-accelerated episodes (410 episodes), VT acceleration occurred in VTs with shorter CL. The mean VT CL in the accelerated episodes was 318.71 ± 25.92 SD compared to a mean of 337.31 ± 52.70 SD in the non-accelerated episodes (*P* value: 0.032). Receiver operating characteristics (ROC) curve was used to define a cutoff value of VT cycle length that predicts VT acceleration. The cutoff value that predicts VT acceleration by ATP therapy was a mean VT CL < 310 ms, with sensitivity of 76.3% specificity of 67.7% positive predictive value of 45% and negative predictive value of 86% with diagnostic accuracy of 0.79, Fig. 1.

**Fig. 2:**
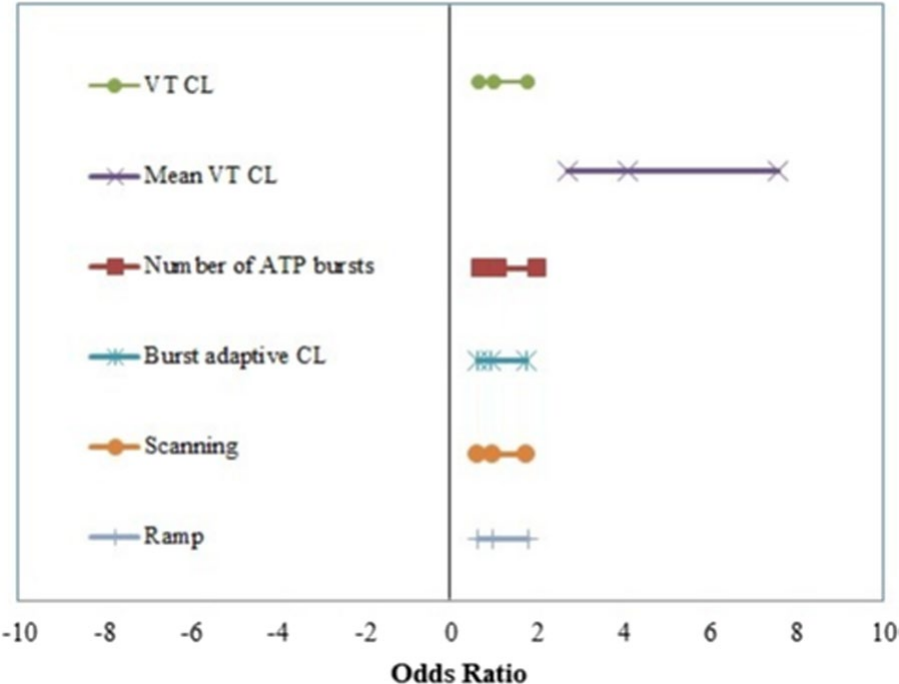
Odds ratio of factors affecting VT accelerated by ATP therapy

ATP programming parameters significantly affected the response to ATP therapy, a greater number of ATP bursts and shorter adaptive cycle length were observed more in the ATP accelerated episodes, and ramp pacing and scanning also were frequently applied in the accelerated episodes as shown in table values, Table 3. Multivariate analysis was performed using logistic regression tests including the following variables: the VT cycle length, the mean VT cycle length, number of ATP bursts, burst adaptive cycle length, scan and ramp. Among the previous parameters, the VT CL, mean VT CL, number of ATP bursts and ramp had a significant effect on VT acceleration by ATP therapy, Fig. 2.

**Table 3:** Comparison between ATP accelerated episodes and non-accelerated episodes according to ATP programming parameters

ATP programming parameters	VT accelerated episodes (*n* = 38)	Non-accelerated VT episodes (*n* = 410)	*P*-value
Number of ATP of bursts			
Mean ± SD	3.66 ± 2.22	1.76 ± 1.35	< 0.001**
Range	1–9	1–11	
Number of stimuli in each burst			
Mean ± SD	8.50 ± 0.98	8.22 ± 1.05	0.113
Range	5–10	5–13	
Burst adaptive CL			
Mean ± SD	83.55 ± 2.92	84.64 ± 2.61	0.016*
Range	80–88	75–88	
Scanning			
No	17 (44.7%)	281 (68.7%)	0.003*
Yes	21 (55.3%)	128 (31.3%)	
Ramp			
No	29 (76.3%)	392 (95.8%)	< 0.001**
Yes	9 (23.7%)	17 (4.2%)	

*ATP* anti-tachycardia therapy, *n* number, *VT* ventricular tachycardia, *SD* standard deviation, *CL* cycle length

Using: t-independent sample t test; χ^2^: Chi-square test. *P*-value > 0.05 NS; *P-value < 0.05 S; **P-value < 0.001 HS

## Discussion

Defibrillation therapy is highly effective in terminating life-threatening ventricular arrhythmia and preventing SCD in the patients who are at high risk [5]. ATP therapy provides a painless adjuvant therapy to the ICD shocks with a comparable efficacy and better quality of life [6]. However, VT acceleration in response to ATP therapy leading to ICD shocks has been observed [7]. In this study we investigated the risk factors of VT acceleration by ATP therapy in 60 patients with structural heart disease who received ATP therapy for terminating monomorphic VT; 448 episodes were reviewed. The percentage of VT accelerated episodes was 8.5% regardless of the underlying structural heart disease; impaired left ventricle ejection predicted patients who had accelerated episodes. Short VT CL, multiple ATP bursts and the use of ramp predicted acceleration of VT.

Previous studies proved ATP efficacy in terminating VT episodes with a low failure rate and low acceleration rate. [6, 8] In the current study, the success rate was 69.86% and acceleration rate was 8.5%. All accelerated episodes were further terminated by subsequent device therapy either an additional ATP therapy or shock. Impairment of the LV ejection fraction was a strong predictor for VT acceleration in this study, but there was no relation between the underlying structural heart disease and the acceleration in response to ATP. This was in agreement with the observation of Hammil et al. [9] who found that patients with higher LV EF showed higher success rate with ATP therapy (both burst and auto-decremental pacing), and patients with lower LV EF are more likely to accelerate with auto-decremental pacing but not burst pacing; also, they found no relation between the underlying structural heart disease and the response to ATP therapy.

Previous studies have suggested the effect of antiarrhythmic drugs on slowing the VT rate by the use of antiarrhythmic, thus facilitating VT termination by ATP therapy [10]. The present study could not demonstrate a difference in the occurrence of VT acceleration and the use of any of the antiarrhythmic drugs. However, the effect of the AAD was not systematically assessed because of the continuously changing types and doses of AAD throughout the time of the retrieved episodes. Peter et al. [11] found that VT termination in response to ATP therapy was less successful in patients receiving class I or class III antiarrhythmic drugs; they attributed that to the effect of antiarrhythmic drugs on prolonging the effective refractory period, thus reducing the excitable gap. However, they did not relate the occurrence of VT acceleration to AAD.

Regarding demographic characteristics, Peter et.al. also found that ATP was less successful in women and that women had almost threefold incidence of VT acceleration when compared with men (14% vs. 5%, *P*: 0.001) and that was independent of other clinical variables as the LV ejection fraction, age, the underlying structural heart disease, NYHA functional class or the use of AAD; however, this observation could not be explained. [11] In the present study, there was no difference in VT acceleration related to gender.

The role of VT cycle length in predicting ATP success or VT acceleration by ATP therapy was investigated by Josephson and colleagues [12], and they reported that the VTCL was the most important factor of ATP terminating reentrant VT. Shorter VTCLs are associated with less possibility of ATP to penetrate the excitable gap of the reentrant circuit and thus terminate the tachycardia which is consistent with data of the present study. Calkins et al. [13], Hammill et al. [9] and Peters et al. [11] reported that the incidence of VT acceleration by ATP therapy in VTCL < 300 ms was higher than with VTCL ≥ 300 ms in a prospective analysis of induced VTs. Nasir et al. [14] found that the mean VT cycle lengths for episodes responding with termination, failure and acceleration averaged 395 ± 65, 390 ± 64 and 347 ± 54 ms, respectively. VT cycle lengths associated with acceleration did not differ between ramp and burst pacing. Fang et al. [15] found a cutoff point of 347 ms in VTCL that predicts ATP acceleration with the sensitivity of 82.1%; VTs > 347 ms are unlikely to be accelerated by ATP attempts.

In the current study we found that both ramp pacing and the higher number of ATP bursts delivered (mean = 3.66 ± 2.22 SD) showed a high statistically significant difference between the accelerated and non-accelerated episodes (*P*: 0.001). Scanning with a scan step -10 ms in all episodes and a lesser adaptive cycle length (mean = 83.55 ± 2.92 SD) showed a statistically significant difference between the accelerated and non-accelerated episodes (*P* value: 0.003) & (*P*: 0.016), respectively. Number of stimuli in each burst did not show any difference in VT acceleration by ATP therapy. In faster VTs with mean VT CL shorter than 310 ms, the number of VT bursts and the number of stimuli in each burst were the most predictors of acceleration followed by scanning and shorter adaptive cycle length. Ramp pacing did not show a statistically significant relation at those rates.

Hammill et al. [9] found that a shorter adaptive cycle length was associated with VT acceleration using both auto-decremental pacing (*P*: 0.03) and burst pacing (*P*: 0.02). Increased number of pulses was associated with acceleration using auto-decremental pacing (*P*: 0.007). Four small prospective randomized studies [13, 16–18] have been undertaken to assess the efficacy of both pacing algorithms (burst & ramp) in VT termination. They found that both are equally effective, and efficacy ranged from 65 to 90%. The incidence of acceleration ranged from 3.7 to 21%. However, the incidence of acceleration was not different between the two modes of pacing in the four studied groups. Nasir et al. [14] found that acceleration rate did not differ between ramp and burst pacing. Adaptive cycle length shorter than 70% was associated with acceleration rate 4%. In the ADVANCE-D trial [7] comparing the efficacy of two different sequences of ATP burst strategies (15 vs. 8 stimuli in each burst) for the termination of fast ventricular tachycardia, they found that fifteen-pulse burst ATP was significantly better in patients without a previous history of heart failure (OR 5.21, 95%CI 1.39–19.50, *P* = 0.014) and in patients with left ventricular ejection fraction (LVEF) ≥ 40% (OR 5.97, 95%CI 1.39–25.62, *P* = 0.016). Eight-pulse ATP was more effective in patients with previously reported heart failure, but only in those with NYHA functional class I–II (OR 0.38, 95%CI 0.16–0.91, *P* = 0.029). Fang Y et al. [15] found that burst pacing with more pulse numbers was more likely to cause VT acceleration, especially in those with cycle length < 347 ms. Table 4 summarizes data addressing effect of ATP therapy on VT acceleration.

**Table 4:** Summary for previous studies addressing VT acceleration in response to anti-tachycardia pacing

Study	Study design	Studied VT episodes	VT acceleration %	Predictors of VT acceleration	ATP success rate	ATP failure rate	AAD	LV EF	Structural heart disease
Fang et al. [15]	Retrospective study	Spontaneous VT & induced VT in EPS in in ICD patients	3.7%	Number of VT morphologies in EGMs, VT induced by EPS, VTCL < 347 ms, mean variation in VTCL, burst stimulation with more pulse numbers	81.43%	14.7%	Not reported	Mean LV EF = 50.5% in the accelerated group; mean LV EF = 59.9% in the non-accelerated group	No relation found between the underlying structural heart disease and ATP success or failure
The SATISFACTION study [19]	Prospective, randomized, multicenter trial	Spontaneous VT in ICD patients	2.9%	Not reported	61.7% Predictors of success: female sex, CAD, primary prevention, narrow QRS, FVT CL, ACEI/ARB and absence of β-blocker	38.3%	Not reported	mean LV EF = 42.9 ± 17.2%	There was no difference between patients with CAD& non-CAD in VT acceleration by ATP
ADVANCE-D Trial [10]	Prospective, parallel and randomized, multicenter trial	Spontaneous VT in ICD patients	3.9%	Not reported	67% Predictors of success: administration of ACEI and NYHA functional class	33%	AAD therapy did not affect the response to ATP therapy	Mean LV EF = 33.9 ± 12.1%	Patients with CAD showed comparable response to ATP therapy as those with no CAD
Pain Free Rx II [6]	Prospective, randomized, multicenter trial	Spontaneous VT in ICD patients	2% in FVT (240–320 ms)	Not reported	72% in FVT No clinical predictors of success were found. With every 5% increase in LV EF the odds of successful ATP for FVT are 18% higher	28% in FVT	Not reported	Mean LVEF = 32 ± 13%	ATP success was 67% in patients with CAD and 83% in patients without CAD (*P* = 0.16)
Pain free Rx I [8]	Prospective multicenter trial	Spontaneous VT in CAD patients	4% in FVT (240–320 ms)	Not reported	85% in FVT	15% in FVT	Not reported	Mean LV EF = 33 ± 13%	54% of patients were on AAD. No conclusion regarding its effect due to limited duration of follow-up
Peters et al. [11]	Retrospective study	Spontaneous VT in ICD patients	6.2%	Female sex aggressive ramp pacing VTCL < 300 ms	77.1%	16.5% Predictor of failure: female sex, history of MI, more severe LV dysfunction, being on class I or III AAD, Ramp pacing	ATP was less successful in patients receiving class I& III AAD	Mean LV EF = 35 ± 14%	CAD was one of the predictors of ATP failure
Nasir et al. [14]	Retrospective study	Spontaneous VT in ICD patients	Less than 2%	Mean VT CL for accelerated episodes = 347 ± 54 ms	94%	6%	Not reported	Mean LV EF = 31 ± 12%	Not reported
Hammil et al. [9]	Non-randomized, retrospective, multicenter study	Induced VT by NIPS in patients with ICD	12% in LV EF < 40%, 9% in LV EF > 40%	Low LV EF, VTCL < 300 ms, increased number of ATP stimuli	49% In EF < 40% and 65% in EF > 40%, predictors of ATP success: higher ejection fraction & Long VVT CL	Not reported	No effect of AAD on tachycardia therapy results	Mean LV EF = 36 ± 15%	No effect of underlying structural heart disease on tachycardia therapy results
Calkins et al. [13]	Prospective study	Induced VT in the EP laboratory	21% with fixed burst pacing 18% with decremental burst pacing	VTCL < 300 ms	70% with fixed burst pacing 72% with decremental burst pacing	9% with fixed burst pacing 10% with decremental burst pacing	No effect of AAD on tachycardia therapy results	Mean LV EF = 34 ± 17%	Not reported

*VT* ventricular tachycardia, *ATP* anti-tachycardia pacing, *AAD* antiarrhythmic drugs, *LV EF* left ventricular ejection fraction, *EPS* electrophysiologic study, *ICD* implantable cardioverter defibrillator, *VTCL* ventricular tachycardia cycle length, *CAD* coronary artery disease, *F VT* fast ventricular tachycardia, *CL* cycle length, *SCD* sudden cardiac death, *NIPS* non-invasive programmed stimulation

## Limitations

(i) Being a non-randomized retrospective study, (ii) ATP programming parameters were empirical upon the treating physicians’ preference which might cause bias. (iii) Due to limited device memory, not all VT episodes were retrieved which might affect data analysis. (iv) The effect of antiarrhythmic medications could not be assessed effectively as they were continuously changed or dose adjusted through the time of the retrieved episodes.

## Conclusions

VT acceleration by ATP therapy is one of the drawbacks of ATP therapy; it occurred in 8.5% of our studied patients. VTs with CL shorter than 310 ms are more likely to accelerate. Short VT CL, high number of ATP bursts, short burst adaptive cycle length, scan and ramp are all predictors of VT acceleration.

Among the previously mentioned predictors of VT acceleration, the VT CL, number of ATP bursts and ramp had a significant effect on VT acceleration. Fast VTs are more likely to be accelerated by burst pacing with a greater number of stimuli in each burst. These findings are mostly compatible with the previous similar studies; it provides an evidence to guide optimizing ATP reprogramming after VT acceleration added to optimizing the medical therapy and the invasive methods as VT ablation.

## Data Availability

The datasets used and/or analyzed during the current study are available from the corresponding author on reasonable request.

## Abbreviations

AAD: Anti-arrhythmic drug
ATP: Anti-tachycardia pacing
Bpm: Beat per minute
CAD: Coronary artery disease
CRT: Cardiac resynchronization therapy
CRT-D: Cardiac resynchronization therapy with defibrillator
ECG: Electrocardiogram
EGM: Electrograms
EPS: Electrophysiologic study
F VT: Fast VT
LV: Left ventricle
LV EF: Left ventricle ejection fraction
MS: Milli second
NIPS: Non-invasive programmed stimulation
ROC: Receiver operating curve
SD: Standard deviation
SCD: Sudden cardiac death
SPSS: Statistical Package for Social Sciences
VT CL: Ventricular tachycardia cycle length
VT: Ventricular tachycardia
VF: Ventricular fibrillation
